# Menopausal factors and risk of seropositive rheumatoid arthritis in postmenopausal women: a nationwide cohort study of 1.36 million women

**DOI:** 10.1038/s41598-020-77841-1

**Published:** 2020-11-27

**Authors:** Yeonghee Eun, Keun Hye Jeon, Kyungdo Han, Dahye Kim, Hyungjin Kim, Jaejoon Lee, Dong-Yun Lee, Jung Eun Yoo, Dong Wook Shin

**Affiliations:** 1 Department of Medicine, Samsung Medical Center, Sungkyunkwan University School of Medicine, Seoul, 06351 South Korea; 2 Department of Family Medicine and Supportive Care Centre, Samsung Medical Center, Sungkyunkwan University School of Medicine, Seoul, South Korea; 3 Department of Statistics and Actuarial Science, Soongsil University, Seoul, South Korea; 4 Department of Obstetrics and Gynecology, Samsung Medical Center, Sungkyunkwan University School of Medicine, Seoul, South Korea; 5 Department of Family Medicine, Healthcare System Gangnam Center, Seoul National University Hospital, Seoul, South Korea; 6 Department of Digital Health, Samsung Advanced Institute for Health Science and Technology (SAIHST), Sungkyunkwan University, Seoul, South Korea; 7 Department of Medical Humanities, Samsung Medical Center, Sungkyunkwan University School of Medicine, Seoul, South Korea

**Keywords:** Rheumatology, Risk factors

## Abstract

In previous literature regarding development of rheumatoid arthritis (RA), female reproductive factors have been described as protective factors, risk factors, or irrelevant, leading to inconsistent results. The aim of this study was to investigate the effect of female reproductive factors on the incidence of seropositive RA. A large population-based retrospective cohort of the National Health Insurance Service data in South Korea was used. Postmenopausal women who participated in both cardiovascular and breast cancer screening in 2009 were included and followed until date of seropositive RA diagnosis, death, or December 31, 2018. Multivariable-adjusted Cox proportional hazards model was used to assess the association between reproductive factors and incident seropositive RA. Of 1,357,736 postmenopausal women, 6056 women were diagnosed with seropositive RA, and the incidence rate was 54.16 cases/100,000 person-years. Reproductive factors other than hormone replacement therapy (HRT) were not significantly associated with seropositive RA incidence. Postmenopausal women who used HRT ≥ 5 years were associated with a higher aHR of incident seropositive RA than never-users (aHR 1.25; 95% CI 1.09–1.44). Alcohol consumption less than 30 g per day (aHR 0.80; 95% CI 0.74–0.87), regular physical activity (aHR 0.90; 95% CI 0.84–0.97), diabetes mellitus (aHR 0.85; 95% CI 0.78–0.93), and cancer (aHR 0.77; 95% CI 0.64–0.92) were associated with lower risk of seropositive RA. Most female reproductive factors did not significantly affect the development of seropositive RA in postmenopausal women. Only HRT is associated with a small but significant increase in risk of seropositive RA.

## Introduction

Rheumatoid arthritis (RA), the most common inflammatory arthritis, can lead to joint destruction, deformity, and functional loss. The global prevalence rate of RA is estimated to be 0.25% according to the Global Burden of Disease 2017 study^1^. RA is more common in women than men, and its incidence and life time risk are about twice as high in women^2,3^. Genetic (e.g. skewed inactivation of X-chromosome), gender-specific hormonal (e.g. estrogens, progesterone, and androgens), and lifestyle factors (e.g. occupational differences) are considered to contribute to the sexual dimorphism of RA^4,5^.

The peak incidence of RA is similar to age at menopause in women, suggesting that lower ovarian function may be associated with risk of RA development^6^. To date, female reproductive factors studied for association with RA include age at menarche^7,8^, age at menopause^9–12^, parity^13–18^, breast feeding^8,19–22^, use of oral contraceptives (OC)^23–28^, and hormonal replacement therapy (HRT)^29,30^. For the development of RA, these factors have been described as protective factors^8,14,18,20–22,24,27–29^, risk factors^9,10,12,15,17,23^, or irrelevant^11,25,26,30^, depending on the study, leading to inconsistent results. Previous literature showed that the effect of parity on RA varies with age^15,18^. These findings suggest that the impact of reproductive factors on RA may vary depending on the stage of reproductive aging.

Many previous studies were conducted in a cross-sectional^9,23^ or case-control^7,10,13,15,18,22,24,27–29^ manner, and the number of patients with RA included in the study was not large enough (most studies are < 1000 cases) to establish an association between reproductive factors and RA development. Some previous studies could not rule out potential confounder effects because only a few reproductive factors were included as variables of interest or other RA risk factors were not adequately controlled^13,16–18,20,27^. In addition, most research has been done in the western region, and published data on the Asian population remains limited^20,23^.

Previous reports have mentioned that reproductive factors can have different impacts in seropositive RA and seronegative RA^12,24,29^. However, many studies did not perform analyses according to seropositivity^7,9,11,13,17–20,23,30^. In Korea, reproductive history is collected through a national health check-up program, and all incident seropositive RA is registered to a special co-payment reduction program with enhanced benefit coverage. Therefore, the aim of this study was to investigate the effect of female reproductive factors on incidence of seropositive RA in a large nationwide population-based cohort of postmenopausal women in South Korea.

## Results

### Baseline characteristics of study participants

Of 3,554,158 female subjects (age ≥ 40 years) who had undergone both cardiovascular and breast cancer screening from 1 January 2009 to 31 December 2009, 1,928,461 eligible postmenopausal women were identified. Among 1,928,461 postmenopausal women, we excluded those with previous RA diagnosis (n = 35,365), disability (n = 109,909), previous hysterectomy (n = 203,854), death (n = 3610), or seropositive RA diagnosis (n = 1074) within 1 year after examination, and those missing data in any variables of interest (n = 216,913). Those who had previously undergone a hysterectomy were excluded because the questionnaire provided no information about hysterectomy timing and whether a bilateral oophorectomy had been performed concurrently. After those exclusions, our analyses finally included a total of 1,357,736 individuals.

Characteristics of the study population are described in Table 1. The mean age of the total study participants was 61.4 years. Overall mean age at menarche and menopause were estimated at 16.5 and 50.0 years, respectively. Among all participants, 91.4% had parity more than 2, 69.3% had breast feeding experience for more than 1 year, 80.9% were never HRT users, and 80.0% were never OC users. Of note, there was no noticeable difference in characteristics between the two study groups (seropositive RA group and non-cases group), and the characteristics of these two groups were comparable to those of total study participants.

**Table 1:** Selected baseline characteristics of study population.

Variable	Total (N = 1,357,736)	Women not diagnosed with seropositive RA (N = 1,351,680)	Women diagnosed with seropositive RA (N = 6,056)	OR (95% CI)	*p*-value
Mean age, y (SD)	61.4 (8.3)	61.4 (8.3)	60.7 (7.6)	0.99 (0.99–0.99)	< 0.001
**Age at menarche, N (%)**					
Mean (SD)	16.5 (1.8)	16.5 (1.8)	16.5 (1.9)	1.01 (0.99–1.02)	0.332
≤ 15 years	422,281 (31.1)	420,445 (31.1)	1836 (30.3)	1 (ref.)	0.474
16 years	287,920 (21.2)	286,618 (21.2)	1302 (21.5)	1.04 (0.97–1.12)	
17 years	269,426 (19.8)	268,189 (19.8)	1237 (20.4)	1.06 (0.98–1.14)	
≥ 18 years	378,109 (27.9)	376,428 (27.9)	1681 (27.8)	1.02 (0.96–1.09)	
**Age at menopause, N (%)**					
Mean (SD)	50.0 (4.0)	50.0 (4.0)	50.0 (4.0)	1.00 (0.99–1.00)	0.499
< 40 years	23,170 (1.7)	23,064 (1.7)	106 (1.8)	1 (ref.)	0.588
40–44 years	77,503 (5.7)	77,131 (5.7)	372 (6.1)	1.05 (0.85–1.30)	
45–49 years	369,193 (27.2)	367,536 (27.2)	1657 (27.4)	0.98 (0.81–1.19)	
50–54 years	743,286 (54.7)	740,016 (54.8)	3270 (54.0)	0.96 (0.79–1.17)	
≥ 55 years	144,584 (10.7)	143,933 (10.7)	651 (10.8)	0.98 (0.80–1.21)	
**Reproductive period, N (%)**					
Mean (SD)	33.6 (4.4)	33.6 (4.4)	33.5 (4.4)	1.00 (0.99–1.00)	0.307
< 30 years	186,341 (13.7)	185,493 (13.7)	848 (14)	1 (ref.)	0.660
30–34 years	567,444 (41.8)	564,895 (41.8)	2549 (42.1)	0.99 (0.91–1.07)	
35–39 years	517,205 (38.1)	514,913 (38.1)	2292 (37.9)	0.97 (0.90–1.05)	
≥ 40	86,746 (6.4)	86,379 (6.4)	367 (6.1)	0.93 (0.82–1.05)	
**Parity, N (%)**					
Nulliparous	33,605 (2.5)	33,429 (2.5)	176 (2.9)	1 (ref.)	0.084
1 child	82,685 (6.1)	82,308 (6.1)	377 (6.2)	0.87 (0.73–1.04)	
≥ 2 children	1,241,446 (91.4)	1,235,943 (91.4)	5503 (90.9)	0.85 (0.73–0.98)	
**Duration of breastfeeding, N (%)**					
Never	90,070 (6.6)	89,617 (6.6)	453 (7.5)	1 (ref.)	0.026
< 0.5 years	89,470 (6.6)	89,089 (6.6)	381 (6.3)	0.85 (0.74–0.97)	
0.5 to < 1 years	237,224 (17.5)	236,135 (17.5)	1089 (18.0)	0.91 (0.82–1.02)	
≥ 1 years	940,972 (69.3)	936,839 (69.3)	4133 (68.3)	0.87 (0.79–0.96)	
**Oral contraceptive use, No. (%)**					
Never used	1,084,881 (79.9)	1,080,038 (79.9)	4843 (80.0)	1 (ref.)	0.477
< 1 years	123,156 (9.07)	122,584 (9.1)	572 (9.5)	1.04 (0.95–1.14)	
≥ 1 years	82,398 (6.07)	82,054 (6.1)	344 (5.7)	0.94 (0.84–1.04)	
Unknown	67,301 (4.96)	67,004 (5.0)	297 (4.9)	0.99 (0.88–1.11)	
**Hormone therapy, N (%)**					
Never used	1,097,847 (80.9)	1,093,086 (80.9)	4761 (78.6)	1 (ref.)	< 0.001
< 2 years	123,195 (9.1)	122,583 (9.1)	612 (10.1)	1.15 (1.05–1.25)	
2 to < 5 years	50,640 (3.7)	50,381 (3.7)	259 (4.3)	1.18 (1.04–1.34)	
≥ 5 years	38,797 (2.9)	38,583 (2.9)	214 (3.5)	1.27 (1.11–1.46)	
Unknown	47,257 (3.5)	47,047 (3.5)	210 (3.5)	1.03 (0.89–1.18)	
**Smoking history, N (%)**					
Never smoker	1,306,285 (96.2)	1,300,499 (96.2)	5786 (95.5)	1 (ref.)	0.024
Former smoker	14,512 (1.1)	14,437 (1.1)	75 (1.2)	1.17 (0.93–1.47)	
Current smoker	36,939 (2.7)	36,744 (2.7)	195 (3.2)	1.19 (1.03–1.38)	
**Alcohol consumption, N (%)**					
Non	1,186,714 (87.4)	1,181,322 (87.4)	5,392 (89.0)	1 (ref.)	0.001
Mild (< 30 g/day)	163,966 (12.1)	163,328 (12.1)	638 (10.5)	0.86 (0.79–0.93)	
Heavy (≥ 30 g/day)	7056 (0.5)	7030 (0.5)	26 (0.4)	0.81 (0.55–1.19)	
Regular physical activity	251,179 (18.5)	250,138 (18.5)	1041 (17.2)	0.91 (0.86–0.98)	0.009
**Anthropometrics**					
Body mass index, N (%)					
< 18.5 kg/m^2^	28,840 (2.1)	28,670 (2.1)	170 (2.8)	1.25 (1.07–1.56)	< 0.001
18.5 to < 23 kg/m^2^	469,107 (34.6)	466,886 (34.5)	2221 (36.7)	1 (ref.)	
23 to < 25 kg/m^2^	360,783 (26.6)	359,175 (26.6)	1608 (26.6)	0.94 (0.88–1.00)	
25 to < 30 kg/m^2^	443,423 (32.7)	441,593 (32.7)	1830 (30.2)	0.87 (0.82–0.93)	
≥ 30 kg/m^2^	55,583 (4.1)	55,356 (4.1)	227 (3.8)	0.86 (0.75–0.99)	
Systolic BP (mmHg)	125.6 ± 16.2	125.6 ± 16.2	124.3 ± 15.8	1.00 (0.99–1.00)	< 0.001
Diastolic BP (mmHg)	76.9 ± 10.2	76.9 ± 10.2	76.4 ± 10.1	1.00 (0.99–1.00)	< 0.001
**Laboratory findings**					
Glucose (mg/dL)	99.7 ± 24.2	99.7 ± 24.3	96.9 ± 21.5	0.99 (0.99–1.00)	< 0.001
Cholesterol (mg/dL)	208.3 ± 43.9	208.3 ± 43.8	205.2 ± 52.3	1.00 (1.00–1.00)	< 0.001
**Comorbidity**					
Hypertension, N (%)	574,745 (42.33)	572,398 (42.4)	2347 (38.8)	0.86 (0.82–0.91)	< 0.001
Diabetes mellitus, N (%)	174,870 (12.88)	174,248 (12.9)	622 (10.3)	0.77 (0.71–0.84)	< 0.001
Dyslipidemia, N (%)	462,410 (34.06)	460,547 (34.1)	1,863 (30.8)	0.86 (0.81–0.91)	< 0.001
Cancer, N (%)	36, 317 (2.7)	36,193 (2.7)	124 (2.1)	0.76 (0.64–0.91)	0.002
**Income**					
Q1 (lowest)	309,432 (22.8)	308,044 (22.8)	1388 (22.9)	1 (ref.)	0.873
Q2	252,891 (18.6)	251,759 (18.6)	1132 (18.7)	1.00 (0.92–1.08)	
Q3	335,670 (24.7)	334,155 (24.7)	1515 (25.0)	1.01 (0.94–1.08)	
Q4 (highest)	459,743 (33.9)	457,722 (33.9)	2021 (33.4)	0.98 (0.92–1.05)	

*OR* odds ratio, *95% CI* 95% confidence interval, *SD* standard deviation, *BP* blood pressure.

### Associations between reproductive factors and risk of seropositive RA

During a mean follow-up of 8.24 years (11,181,236 person-years) after 1 year of lag period, 6056 women were diagnosed with seropositive RA, and the incidence rate was 54.16 cases per 100,000 person-years. Among all examined reproductive factors, only history of HRT (not age at menarche and menopause, number of children, duration of breastfeeding, and use of OC) showed a statistically significant association with the risk of seropositive RA (Table 2). Compared with the never HRT group, all HRT user groups had increased risk of seropositive RA (adjusted hazard ratio [aHR] 1.11 (95% CI 1.01–1.20) in HRT < 2 years, aHR 1.14 (95% CI 1.01–1.30) in 2 years ≤ HRT < 5 years, and aHR 1.25 (95% CI 1.09–1.44) in HRT ≥ 5 years), indicating higher risk with longer use of HRT. Furthermore, similar associations were observed in Model 3 (Table 2).

**Table 2:** Hazard ratios (HR) and 95% confidence intervals (CI) for association between reproductive factors and risk of seropositive rheumatoid arthritis among postmenopausal women.

	Case no.	Duration (person-years)	IR per 100 000 person-years	HR (95% CI)		
				Model 1 (univariate)	Model 2 (multivariable^a^)	Model 3 (multivariable^b^)
**Age at menarche**						
≤ 15	1836	3,486,901.2	52.7	1 (ref.)	1 (ref.)	
16	1302	2,371,367.7	54.9	1.04 (0.97–1.12)	1.07 (0.99–1.15)	
17	1237	2,217,704.0	55.8	1.06 (0.99–1.14)	1.10 (1.02–1.18)	
≥ 18	1681	3,105,263.2	54.1	1.03 (0.96–1.10)	1.07 (1.00–1.15)	
**Age at menopause**						
< 40	106	188,808.6	56.1	1 (ref.)	1 (ref.)	
40–44	372	633,034.4	58.8	1.05 (0.84–1.30)	1.04 (0.84–1.29)	
45–49	1657	3,040,776.6	54.5	0.97 (0.80–1.18)	0.96 (0.79–1.16)	
50–54	3270	6,125,361.4	53.4	0.95 (0.78–1.15)	0.95 (0.79–1.16)	
≥ 55	651	1,193,255.0	54.6	0.97 (0.79–1.19)	1.01 (0.82–1.24)	
**Reproductive period (years)**						
< 30	848	1,523,258.2	55.7	1 (ref.)		1 (ref.)
30–34	2549	4,667,110.4	54.6	0.98 (0.91–1.06)		0.98 (0.90–1.05)
35–39	2292	4,275,185.6	53.6	0.96 (0.89–1.04)		0.96 (0.89–1.04)
≥ 40	367	715,681.9	51.3	0.92 (0.82–1.04)		0.95 (0.84–1.07)
**Parity**						
Nulliparous	176	276,955.1	63.5	1 (ref.)	1 (ref.)	1 (ref.)
1 child	377	682,989.3	55.2	0.87 (0.72–1.04)	0.90 (0.74–1.08)	0.89 (0.74–1.08)
≥ 2 children	5503	10,221,291.7	53.8	0.85 (0.73–0.99)	0.92 (0.78–1.09)	0.92 (0.78–1.09)
**Duration of breastfeeding**						
Never	453	743,663.5	60.9	1 (ref.)	1 (ref.)	1 (ref.)
< 0.5 years	381	740,618.8	51.4	0.84 (0.74–0.97)	0.86 (0.75–0.99)	0.86 (0.74–0.99)
0.5 to < 1 years	1089	1,960,413.0	55.6	0.91 (0.82–1.02)	0.95 (0.85–1.08)	0.96 (0.85–1.08)
≥ 1 years	4133	7,736,540.7	53.4	0.88 (0.80–0.97)	0.95 (0.85–1.06)	0.95 (0.85–1.07)
**Oral contraceptive use**						
Never used	4843	8,926,291.4	54.3	1 (ref.)	1 (ref.)	1 (ref.)
< 1 years	572	1,020,167.2	56.1	1.03 (0.95–1.13)	1.03 (0.94–1.12)	1.03 (0.94–1.12)
≥ 1 years	344	681,743.3	50.5	0.93 (0.83–1.04)	0.94 (0.84–1.04)	0.94 (0.84–1.05)
Unknown	297	553,034.2	53.7	0.99 (0.88–1.11)	0.99 (0.87–1.12)	0.99 (0.87–1.12)
**Hormone therapy**						
Never used	4761	9,023,919.2	52.8	1 (ref.)	1 (ref.)	1 (ref.)
< 2 years	612	1,025,567.6	59.7	1.13 (1.04–1.23)	1.11 (1.01–1.20)	1.11 (1.02–1.20)
2 to < 5 years	259	421,442.2	61.5	1.16 (1.03–1.32)	1.14 (1.01–1.30)	1.14 (1.01–1.30)
≥ 5 years	214	322,718.2	66.3	1.26 (1.10–1.44)	1.25 (1.09–1.44)	1.25 (1.09–1.44)
Unknown	210	387,588.9	54.2	1.03 (0.89–1.18)	1.04 (0.89–1.20)	1.04 (0.89–1.20)
**Smoking**						
Never smoker	5786	10,766,679.6	53.7	1 (ref.)	1 (ref.)	1 (ref.)
Former smoker	75	117,707.9	63.7	1.19 (0.94–1.49)	1.24 (0.98–1.55)	1.23 (0.98–1.55)
Current smoker	195	296,848.6	65.7	1.22 (1.06–1.41)	1.26 (1.09–1.46)	1.26 (1.09–1.46)
**Alcohol consumption**						
Non	5392	9,763,085.0	55.2	1(ref.)	1(ref.)	1 (ref.)
Mild (< 30 g/day)	638	1,359,856.5	46.9	0.85 (0.78–0.92)	0.80 (0.74–0.87)	0.80 (0.74–0.87)
Heavy (≥ 30 g/day)	26	58,294.5	44.6	0.81 (0.55–1.19)	0.72 (0.49–1.06)	0.72 (0.49–1.06)
**Regular physical activity**						
No	5015	9,098,471.2	55.1	1 (ref.)	1 (ref.)	1 (ref.)
Yes	1041	2,082,764.9	50.0	0.91 (0.85–0.97)	0.90 (0.84–0.96)	0.90 (0.84–0.97)
**Body mass index (kg/m**^**2**^**)**						
< 18.5	170	227,153.7	74.8	1.30 (1.11–1.52)	1.29 (1.10–1.51)	1.29 (1.10–1.51)
18.5 to < 23	2221	3,852,584.0	57.7	1 (ref.)	1 (ref.)	1 (ref.)
23 to < 25	1608	2,980,305.2	54.0	0.94 (0.88–1.00)	0.96 (0.90–1.02)	0.96 (0.90–1.02)
25 to < 30	1830	3,663,588.8	50.0	0.87 (0.81–0.92)	0.91 (0.85–0.97)	0.91 (0.92–0.85)
≥ 30	227	457,604.3	49.6	0.86 (0.75–0.99)	0.93 (0.81–1.06)	0.93 (0.81–1.07)
**Hypertension**						
No	3709	6,485,930.5	57.2	1 (ref.)	1 (ref.)	1 (ref.)
Yes	2347	4,695,305.6	50.0	0.87 (0.83–0.92)	0.95 (0.90–1.01)	0.95 (0.90–1.01)
**Diabetes mellitus**						
No	5434	9,769,798.4	55.6	1 (ref.)	1 (ref.)	1 (ref.)
Yes	622	1,411,437.6	44.1	0.79 (0.73–0.86)	0.85 (0.78–0.93)	0.85 (0.78–0.93)
**Dyslipidemia**						
No	4193	7,372,120.1	56.9	1 (ref.)	1 (ref.)	1 (ref.)
Yes	1863	3,809,116.0	48.9	0.86 (0.81–0.91)	0.90 (0.85–0.95)	0.90 (0.85–0.95)
**Cancer**						
No	5932	10,892,011.0	54.5	1(ref.)	1(ref.)	1(ref.)
Yes	124	289,225.0	42.9	0.79 (0.66–0.94)	0.77 (0.64–0.92)	0.77 (0.64–0.92)
**Income**						
Q1 (lowest)	1388	2,544,940.6	54.5	1 (ref.)	1 (ref.)	1 (ref.)
Q2	1132	2,085,588.4	54.3	1.00 (0.92–1.08)	1.00 (0.92–1.08)	1.00 (0.92–1.01)
Q3	1515	2,771,531.0	54.7	1.00 (0.93–1.08)	1.01 (0.94–1.09)	1.01 (0.94–1.09)
Q4 (highest)	2021	3,779,176.2	53.5	0.98 (0.92–1.05)	0.99 (0.92–1.08)	1.00 (0.94–1.08)

*IR* incidence rate, *HR* hazard ratio, *CI* confidence interval.

^a^Adjusted for age, age at menarche and menopause, parity, duration of breastfeeding, duration of hormone therapy, duration of oral contraceptive use, alcohol consumption, smoking, regular physical activity, income, body mass index, hypertension, diabetes mellitus, dyslipidemia, and cancer.

^b^Adjusted for age, reproductive period, parity, duration of breastfeeding, duration of hormone therapy, duration of oral contraceptive use, alcohol consumption, smoking, regular physical activity, income, body mass index, hypertension, diabetes mellitus, dyslipidemia, and cancer.

### Other factors associated with seropositive RA

Compared to never smokers, former smoker (aHR 1.24; 95% CI 0.98–1.55) and current smokers (aHR 1.26; 95% CI 1.09–1.46) had approximately 25% higher risk of seropositive RA (Table 2). Conversely, alcohol consumption of less than 30 g/day (aHR 0.80; 95% CI 0.74–0.87), regular physical activity (aHR 0.90; 95% CI 0.85–0.96), diabetes mellitus (aHR 0.85; 95% CI 0.78–0.93), and cancer (aHR 0.77; 95% CI 0.64–0.92) were associated with lower risk of seropositive RA (Table 2).

## Discussion

Our study demonstrated the effects of female reproductive factors on the incidence of seropositive RA in a large nationwide-population based cohort of postmenopausal women in South Korea. Most female hormonal factors, including age at menarche, age at menopause, parity, breast feeding, and use of OC, were not associated with the risk of seropositive RA. Only HRT was associated with a slightly higher risk of seropositive RA.

In the current study, both age at menarche and age at menopause had no significant impact on development of seropositive RA, consistent with findings of the French early arthritis ESPOIR cohort study and a Norwegian cohort study of subjects participating in breast cancer screening^19,31^. There are conflicting studies, such as the Nurses’ Health Study (NHS), in which early menarche is associated with increased risk of seropositive RA^8^, and Sweden’s nested case–control study or Denmark’s case–control study, which are associated with reduced risk of seropositive RA^7,10^. However, recent cross-sectional studies based on data from the National Health and Nutrition Examination Survey of the United States and South Korea show no correlation between menarche age and RA^9,23^. Recent studies support our results that there is no link between menarche age and seropositive RA.

Similarly, prior studies on the effect of age at menopause on RA also showed contradictory results. Early menopause has been described to increase the risk of RA in several studies^9–12^, but the results of NHS showed that early menopause did not act as a risk factor for seropositive RA^12^. Pikwer et al. also showed that early menopause is associated with high risk of seronegative RA, but there was no significant association with seropositive RA^10^. Most previous studies in which early menopause showed a positive association with RA risk were conducted in a cross-sectional manner and did not classify RA according to seropositivity. Our study confirms the results of the NHS that seropositive RA has little association with menopausal age.

Our study showed that the HR of seropositive RA tended to decrease slightly with parity, but it was not statistically significant. This finding is congruous with the results of previous studies in which there was no association between parity and RA in postmenopausal women^9,11,20^. Conversely, in research including women of childbearing age, protective effects of parity have been reported, suggesting that the effect of parity may vary with age^13,17,18^.

Breastfeeding was also associated with insignificantly lower risk of seropositive RA. This may be interpreted in the same context as the results of previous studies showing a negative association between lactation and RA incidence that was not maintained when confounders were adjusted^10,24^. In two nested case–control studies of the same population in Sweden's Malmo Diet and Cancer Study, statistical significance differed depending on reproductive factors included in the study^10,22^. These results suggest the possibility of interactions between reproductive factors and indicate that the protective effect of breastfeeding is less significant when various reproductive factors are considered together. In our study, the non-significant association between breastfeeding and seropositive RA is thought to be due to inclusion of various reproductive factors in the analyses.

Our findings showed that the use of OC does not affect postmenopausal development of seropositive RA. Some studies have suggested that OC use, especially long-term OC use, is associated with a lower risk of RA^24,28^, but other studies have found that OC use is associated with a higher risk of RA^7,23^. Two meta-analyses published in 2014 failed to demonstrate the protective role of OC in RA development^25,26^, in line with the results of our study.

Unlike endogenous hormonal factors, HRT was associated with increased risk of seropositive RA in this study, and the strength of the association was small but significant. This finding is in line with the results of the NHS that long-term HRT use was associated with an increased risk of seropositive RA^12^ and the results of the Iowa Women’s Health Study that former HRT use was positively associated with RA^11^. Other studies have reported different results. A population-based case–control study in Sweden demonstrated the protective effect of HRT, but reduced risk of RA was only observed in women aged 50–59 years and in the group with combined use of estrogen and progestogen^29^. However, most prior research did not demonstrate a clear link between RA and HRT^7–9,23,27^, including post-hoc analysis of Women’s Health Initiative randomized controlled trials conducted to uncover the effects of HRT^30^.

There are several possible explanations for our findings. In mice experiments, chronic 17β-estradiol administration has been shown to promote production of TLR-4-triggered proinflammatory mediator^32,33^. These results suggest that prolonged exposure to exogenous estrogen contained in HRT played a proinflammatory role and contributed to the development of RA. However, it is possible that the results of our research are not due to the biological effects of estrogen. More than half of women complain of musculoskeletal pain during menopause^34^. Menopausal arthralgia and early RA are often difficult to distinguish^35^. Therefore, postmenopausal women with undiagnosed joint symptoms are more likely to use HRT and RA is diagnosed later when other symptoms and signs became more evident^36^. Considering the results of recent studies that estrogen plays an anti-inflammatory role by increasing IgG sialylation^37^, the latter is a more plausible way to interpret our findings.

Our study also identified an association between demographic and lifestyle factors and the incidence of seropositive RA. The association of lower socioeconomic status and cigarette smoking with higher risk of RA has already been documented in previous reports and has also been reconfirmed in our study^38,39^. As in the preceding literature, mild alcohol consumption and regular physical activity significantly lowered the risk of seropositive RA^40,41^.

Co-morbidities of diabetes and cancer were related to lower risk of seropositive RA. This is in agreement with a recent case–control study conducted in Greece demonstrating low prevalence in patients with type 2 diabetes^42^ and a nationwide population-based cohort study in Taiwan showing the relationship between female breast cancer and lower RA risk^43^. The mechanism by which the risk of RA is lowered in cancer patients is not clear, but the high activity of regulatory T cells seen in cancer patients may contribute^44–46^. The association between well-known risk factors and seropositive RA was also reproduced in our study, suggesting that patients with seropositive RA were properly classified in our study.

To the best of our knowledge, this is the largest study so far to assess the association between female reproductive factors and incidence of seropositive RA. The strengths of our research are inclusion of a sufficient number of seropositive RA cases through a large nationwide population-based cohort and evaluation of the link between risk factors and incidence of seropositive RA using longitudinal follow-up data. We tried to minimize the effects of potential confounders by including various reproductive factors and known risk factors of RA in the analyses. There is a lack of data in the Asian population, and our study provides important information about risk factors of seropositive RA in the Asian population.

There are several limitations of this study. First, reproductive factors and comorbidities were collected by self-reporting, so recall bias could not be completely ruled out. Second, since there was no information on the type, dose, and onset of exogenous hormone preparations, detailed analyses of OC and HRT were not performed. In Korea, a progesterone only pill is used only as a temporary postcoital contraceptive, and all other OCs on the market are combined pills. Therefore, most of the OC users included in this study are estimated to be users of combined pills. Third, because our study focuses only on seropositive RA in postmenopausal women, it is difficult to generalize the findings to seronegative RA or other populations, such as women of childbearing age or men. Since only seropositive RA is included in the registry of intractable diseases (RID), and the validity of defining seronegative RA in the claim database is relatively low, our study excluded seronegative RA. However, previous studies have shown that reproductive factors may differently affect seropositive RA and seronegative RA, and it is reasonable to analyze groups separately according to seropositivity.

In conclusion, most female reproductive factors did not significantly affect development of seropositive RA in postmenopausal women. Only HRT was associated with a small but significant increase of risk of seropositive RA.

## Methods

### Data source and study setting

In South Korea, approximately 97% of the population is covered by universal insurance provided by a single insurer, the Korean National Health Insurance Service (NHIS). The government-financed Medical Aid program that is also administered by the NHIS covers the remaining 3% of the population in the lowest income bracket.

The NHIS provides free biennial national health check-up programs, including a general health examination focused on cardiovascular risk factors, for all citizens aged 40 and above and all employees regardless of age. In addition, the NHIS provides a biennial breast cancer screening program beginning at age 40.

The NHIS keeps almost all medical data, including diagnostic codes, procedures, prescription drugs, and a registry of cancer and rare intractable diseases. It also has beneficiary information and health check-up results. NHIS data were used in previous epidemiologic studies investigating health risk associated with reproductive factors^47^, and details of the database profiles are described in previous literature^48–50^.

The present study protocol conformed to the ethical guidelines of the 1975 Declaration of Helsinki, as revised in 1983. The Institutional Review Board of Samsung Medical Center approved this study (IRB File No. SMC 2019-07-045). The IRB has approved a waiver of the requirement to obtain informed consent from research participants.

### Data collection

Information on health-related behaviors and the menstrual and reproductive histories of participants was obtained using a self-administered questionnaire. According to the National Cancer Screening Program (NCSP) guidelines, women were asked about age at menarche and age at menopause as continuous variables. For analysis, age at menarche was categorized as ≤ 15 years, 16 years, 17 years, and ≥ 18 years, and age at menopause was categorized as < 40 years, 40–44 years, 45–49 years, 50–54 years, and ≥ 55 years. The reproductive period was calculated as the interval between age at menarche and age at menopause. Information on parity number, total lifetime breastfeeding history, HRT history, and use of OC was also collected as categorical variables: parity number (0, 1, or ≥ 2 children), total lifetime breastfeeding history (never, < 0.5 years, 0.5–1 year, and ≥ 1 year), duration of HRT (never, < 2 years, 2–5 years, ≥ 5 years, and unknown), and duration of OC use (never, < 1 year, ≥ 1 year, and unknown). Smoking status was classified as never smoker, former smoker, and current smoker. Drinking was divided into three levels: non, mild (< 30 g of alcohol/day), and heavy (≥ 30 g/day) drinking. Regular physical activity was defined as moderate physical activity for more than 30 min and more than 5 days per week over the past week. Body mass index (BMI) was calculated using weight (kg) divided by height in meters squared (m^2^) and was classified as low (< 18.8 kg/m^2^), normal to overweight (18.5–24.9 kg/m^2^), or obese (≥ 25 kg/m^2^). Hypertension, diabetes, dyslipidemia, and cancer were defined using physician diagnoses or the use of medication, based on self-reporting. Household income was categorized into quartiles based on insurance premium levels (in Korea, insurance premiums are determined by income level), with those covered by Medical Aid (poorest 3%) merged into the lowest income quartile.

### Study outcomes and follow-up

The primary endpoint of this study was newly diagnosed seropositive RA. The NHIS has a special co-payment reduction program for cancer and some intractable diseases to enhance benefit coverage; seropositive RA is an intractable disease covered by this program^51^. In this study, newly diagnosed seropositive RA was identified based on International Classification of Diseases, 10th Revision (ICD-10) codes for seropositive RA (M05) and the seropositive RA registration code (V223) in the RID. Registration in the RID requires positive result of rheumatoid factor test and an official report from a doctor documenting that the patient fulfills classification criteria of RA; this is more valid than using ICD-10 code alone. A previous study has demonstrated that diagnosis in the RID database had higher validity than other insurance data^52^. The cohort was followed from 1 year after health check-up date to the date of incident seropositive RA, death, or until the end of the study period (31 December 2018), whichever came first.

### Statistical analysis

Continuous variables were presented as means ± standard deviation, and categorical variables were presented as numbers and percentages. The incidence rates of seropositive RA were calculated by dividing the number of incident cases by the total follow-up period. Hazard ratios (HRs) and 95% confidence interval (CI) values for seropositive RA were analyzed using the Cox proportional hazards model for various reproductive factors. A multivariable-adjusted proportional hazards model was applied: (1) Model 1 was non-adjusted; (2) Model 2 was adjusted for age, age at menarche and menopause, parity, duration of breastfeeding, duration of HRT, duration of OC use, alcohol consumption, smoking, regular physical activity, income, body mass index, hypertension, diabetes mellitus, dyslipidemia, and cancer; (3) Model 3 was adjusted for reproductive period instead of age at menarche and menopause separately. Complete case analysis was performed except for 6% of cases in which variables of interest were missing. Statistical analyses were performed using SAS version 9.4 (SAS Institute Inc., Cary, NC, USA), and a P value < 0.05 was considered statistically significant.

## Data Availability

The data that support the findings of this study are available from NHIS. Restrictions apply to the availability of these data, which were used under license for this study. Data are available at https://nhiss.nhis.or.kr with the permission of NIHS.
